# *Tenebrio molitor* Larvae-Based Magnetic Polyurea Employed as Crude Oil Spill Removal Tool

**DOI:** 10.3390/ma15145063

**Published:** 2022-07-20

**Authors:** Mostafa Aboelkheir, Fernando Gomes, Cintia Meiorin, Tiago Galdino

**Affiliations:** 1 Programa de Engenharia Civil, Universidade São Judas Tadeu, Rua Taquari 546, São Paulo 03166-000, Brazil; 2 Instituto de Macromoléculas, Professora Eloisa Mano, Centro de Tecnologia-Cidade Universitária, Av. Horacio Macedo, 2030, Bloco J. Universidade Federal de Rio de Janeiro, Rio de Janeiro 21941-598, Brazil; fernando_gomes@ima.ufrj.br; 3 Instituto de Investigaciones en Ciencia y Tecnología de Materiales (INTEMA), Universidad Nacional de Mar del Plata (UNMdP)—CONICET, Mar del Plata 7600, Argentina; cintia.meiorin@gmail.com; 4 UNIGRANRIO—Universidade do Grande Rio Professor José de Souza Herdy, Rio de Janeiro 25071-202, Brazil; galdinotiago241@gmail.com

**Keywords:** magnetic nanocomposites, magnetite nanoparticles, magnetic force test, oil spills sorber, environmental remediation

## Abstract

Renewable resources constitute an extremely rich and varied set of molecules and polymers produced by natural biological activities. Within the applications of these polymers, a very important application is the use of these materials as a sorber for oils or oil spills. The advantage of these nanocomposites is the fact that they integrate different component materials and their properties into a single component material. They have several applications, ranging from environmental remediation to the development of advanced medical applications. This work proposed using magnetic polyurea composites based on an animal substrate from *Tenebrio molitor* larvae to perform oil spill clean-up operations under a magnetic field in the presence of 1% and 3% of magnetite to be tested as magnetic crude oil sorber. The obtained materials were characterized by Fourier transform infrared (FTIR) spectroscopy, X-ray Diffraction (XRD), Thermogravimetric Analysis (TGA), Scanning Differential Calorimetry (DSC), and Low-Field Nuclear Magnetic Resonance (LF-NMR 1H). The sorber material is simple to prepare and inexpensive. The use of magnetite as a magnetic charge allowed for the efficient removal of oil from water with about 28 g of oil per gram of sorber. These results are very promising and encouraging for future environmental recovery studies involving magnetite and sustainable polymers.

## 1. Introduction

In recent decades, efforts to obtain materials from natural resources have increased in industrial applications for solving environmental problems, waste disposal, and the depletion of non-renewable resources [1,2,3,4,5]. Renewable resources constitute an extremely rich and varied set of molecules and macromolecules produced by natural biological activities. In the specific context of the production of bio-derived polymers from renewable resources, the most widely used include vegetable oils, polysaccharides (mainly cellulose and starch), and proteins [6,7,8]. Due to their polar and reactive macromolecular structure, proteins have attracted much attention as possible sources of new polymeric materials [9,10]. Within the applications of these polymers, a very important application is the use of these materials as a sorber for oil or oil spills.

Recently, our research group reported the biodegradative activity of mealworms of *Tenebrio molitor* Linnaeus larvae on rubber samples as the solo meal [11]. Such a property is trending and has also been investigated by numerous researchers on various plastic molecules over the past few years [12,13,14,15,16,17,18,19]. As a principle of circular economy and sustainability, the authors of this manuscript decided to reuse the same larvae applied in consuming the rubber samples as a protein source to synthesize biomolecules to be tested as oil absorbers.

A strategy to improve the properties of polymeric materials is the addition of a filler such as nanoclays, nano-oxides, cellulose nanocrystals, and organic fillers, among others, to obtain a new composite material [20,21,22]. The use of magnetic nanoparticles in different polymers has received great attention in recent years due to their non-toxic nature, biocompatibility, and the multiple applications that can be derived from the use of these materials, as well as the ease of their preparation [23,24,25]. Among them, magnetic polymer nanocomposites can be defined by the presence of a polymer matrix (organic) and a magnetic (inorganic) compound of nanometric size (e.g., nanoparticles) [26]. The advantage of these nanocomposites is the fact that they integrate different component materials and their properties into a single component material. The final properties depend on the filler size and the distribution of nanoparticles into the matrix, as well as nanoparticle–matrix interactions [27]. They have several applications, ranging from environmental remediation to the development of advanced medical applications. In this work, the use of magnetite as a magnetic filler allowed the efficient removal of oil from water. A magnetic force test that can be conducted at a lower cost compared to instrumental highly sensitive superconductive magnetometers (HTS-SQUID) was reported by our group to study the in situ polymerization of lactic acid in the presence of different amounts of maghemite [28].

The Biopolymers and Sensors Laboratory has been working on environmental recovery since 2010 when our group developed magnetizable foams based on glycerin from castor oil [29] and alkyd resin and cured with TDI [30]. The oil removal capability per gram of resin (ORC) of these materials was equal to 4 g/g and 8 g/g, respectively. In 2012 three new resins were produced. The first was based on cashew nut shell liquid (CNSL), furfural, and lignin [31] and served as proof of the relevance of chemical similarity in the oil removal process, measured by root mean square error (RMSE) analysis among the petroleums and resins measured by FTIR spectra. The other two magnetizable resins were made using cardanol and furfural [32], as well as CNSL, lignin, and formaldehyde [33] from an ORC equal to 10 g/g and 11 g/g, respectively. The insertion of plant fillers began to be studied in 2014 when we prepared a magnetic resin composed of cardanol, furfural, and acetylated curauá fibers [34]. This material presented an ORC equal to 12 g/g. Coffee grounds were used as a filler for a magnetizable and crosslinked polyester resin in 2015 [35] leading to an ORC value equal to 25 g/g. A new magnetizable polyurethane resin based on glycerin and castor oil [36] with an ORC equal to 10 g/g was prepared by emulsion in 2017. Although with a lower ORC, this resin opened a new chapter in our research activities, allowing the direct production of particulates in a heterogeneous medium. In 2018 we produced a new green polyurethane loaded with coffee grounds before and after acetylation [37]. The ORC of both materials was about 9.5 g/g. However, water sorption decreased by 50% when the composite was filled with acetylated coffee. Also in 2018, we prepared a new magnetizable alkyd resin based on phthalic anhydride, castor oil, and glycerin [38]. This preparation was optimized via experimental design and the ORC was equal to 20 g/g. A second magnetizable alkyd resin was loaded with lignin [39] and showed an ORC of 25 g/g. In 2018, urea-loaded poly(butylene succinate) was used as a bioremediation system [40], and then in 2019, a magnetizable version of this polymer [41] showed an ORC equal to 11 g/g. Finally, in 2021 surface-modified magnetite was produced and tested, showing an ORC of 28 g/g [42], and an extrinsically magnetizable porous geopolymer presented an intrinsic oil removal capability (IORC) of 150 g/g [43].

Therefore, this work proposed using magnetic polyurea composites based on an animal substrate from *Tenebrio molitor* larvae to perform oil spill clean-up operations under a magnetic field in the presence of 1% and 3% of magnetite, to be tested as a magnetic crude oil sorber. The results showed that one gram of the material removes about 28 g of oil per gram of sorber.

As this is the first time this material has been presented, there is no further evidence regarding its sustainable nature. On the other hand, as the material is produced based on animal protein from low-cost larvae, which have already been used in the decomposition of polymeric samples [11], the material’s production costs are neglectable, leading to the conclusion that the prepared material is auspicious to environmental recovery applications.

Although producing reliable magnetic results is one of the top priorities for researchers dealing with magnetic nanocomposites, other characterization techniques need to be conducted to assure the formation of the desired materials/nanocomposites. The tested materials were prepared by in situ polymerization of polyurea in the presence of 1% and 3% of magnetite to be tested as a magnetic crude oil sorber. The obtained materials were characterized by Fourier transform infrared (FTIR) spectroscopy, X-ray Diffraction (XRD), Thermogravimetric Analysis (TGA), Scanning Differential Calorimetry (DSC), and Low-Field Nuclear Magnetic Resonance (LF-NMR 1H).

## 2. Experimental

### 2.1. Materials

**Figure 1:**
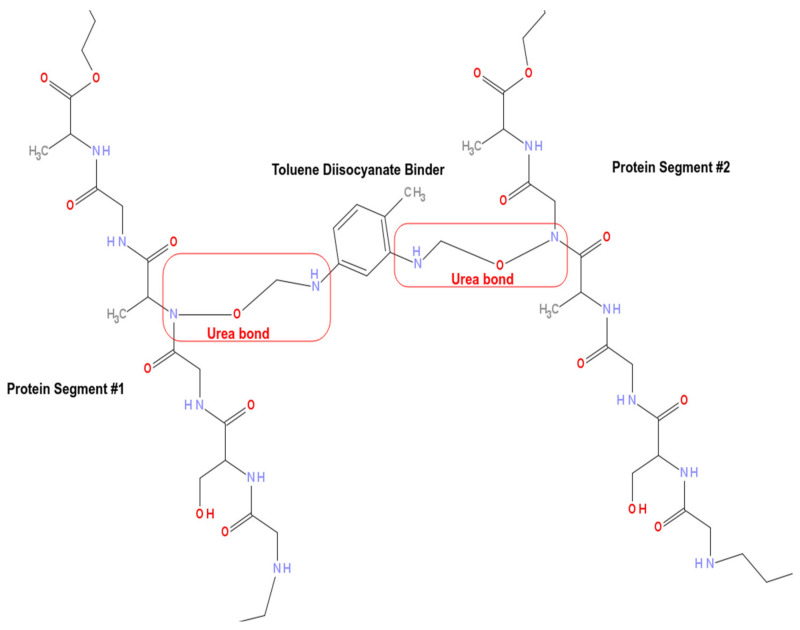
Two protein segments. Each one of the protein segments is composed of -Gly-Ser-Gly-Ala-Gly-Ala- fragments and between these two fragments, there is a TDI binder connecting the protein fragments through two ureic bonds.

Nanoparticles of magnetite were synthesized in our laboratory using the preparation method described in [28]. Polyurea (see Figure 1) was synthesized by the reaction between larvae protein extract and Toluene Diisocyanate (TDI) in addition to the crude oil samples. All the applied chemical reagents were purchased from Vetec/Brazil as analytical-grade, where they were used for syntheses and tests as received without further purification.

### 2.2. Methods

#### 2.2.1. Polyurea Synthesis

The polyurea was prepared following a factorial plan, where different weights of protein were mixed with toluene diisocyanate (TDI) and later, the magnetite was added in situ to prepare the composite. The materials selected for the petroleum sorption tests contained 1% and 3% magnetite. Five repetitions were prepared using the same procedure with different amounts of polyurea with the addition of a magnetic charge in the polar phase to serve as a sorber and determine its intrinsic oil sorption capacity.

#### 2.2.2. Magnetic Force Test

Magnetic force tests were performed using a homemade experimental setup, as described in [28]. This setup constituted an analytical balance Shimadzu AY-220, a voltage source ICEL PS-4100, a digital multimeter ICEL MD-6450, a gaussmeter GlobalMag TLMP-Hall-02, a homemade sample holder, and a homemade electromagnet. System calibration was performed in the absence of magnetic material. First, using the amperemeter and the gaussmeter, a current versus magnetic field calibration was performed. Afterward, a current versus mass calibration was also performed. Obtained results were used to predict some of the presented errors. Magnetic force tests were performed following the mass variation of the sample in the presence of the magnetic field produced by the electromagnet. Then, the apparent variation of mass of the sample in the presence of a magnetic field was calculated by subtracting the mass of the sample in the presence of a magnetic field from the mass of the sample. The magnetic force (opposite to the gravitational one) was calculated according to Equation (1),

(1) $${\mathrm{Fm}}_{n}=\frac{\Delta m\;\times \;g}{m_{0}}$$

where Fm_n_ is the magnetic force normalized by the initial mass of the sample m_0_, Δm is the apparent variation of mass in the presence of the magnetic field, and g is the acceleration of gravity.

#### 2.2.3. Crude Oil Magnetic Removal

These tests were performed at room temperature using a synthetic brine. The brine was prepared using sodium chloride and calcium chloride. The crude oil used in this work presented a density equal to 0.9730 g/mL and °API (@60 °F) equal to 13. A 100 mL beaker containing 90 mL of brine was used into which 0.5 g of crude oil was spilled. After that, a known weight of the absorber was added to the crude oil spot. The beaker was left for 5 min for the composites to interact with the crude oil and form a semi-solid paste that could be removed using a magnet. The crude oil amount (Or) removed from the water was determined by gravimetry using Equation (2) [39],

(2) $$\mathrm{Or}=\frac{w_{2}-w_{3}}{w_{1}}$$

where w_1_ is the composite weight, w_2_ is the total weight (beaker with water and crude oil), and w_3_ is the system weight after removal (beaker with water and residual crude oil). This method allows obtaining the ratio between the removed crude oil and the composite (g/g).

#### 2.2.4. X-ray Diffraction (XRD)

X-ray diffraction measurements were performed using a shimadzu model DRX-6000 (Shimadzu, Kyoto, Japan) at the Instituto de Macromolecules Proessora Eloisa Mano, Rio de Janeiro, Brazil. Under normal temperature and atmospheric pressure conditions, the equipment works with a copper source (Cu Kα = 0.154 nm) under 40 kV and 20 mA. The crystalline size (Lc) was calculated using Scherrer’s Equation (Equation (3)) [44].

(3) $$\frac{K\;\times \;\lambda }{\beta \;\times \;\cos\theta }$$

where Lc is the crystalline size, λ is the wavelength, β is the half-width, and *θ* is the diffraction angle.

#### 2.2.5. Fourier Transform Infrared Spectroscopy Using Attenuated Total Reflectance (FTIR-ATR)

FTIR–ATR analyses of the samples’ powders were performed in a Perkin–Elmer 1720X Fourier transform spectrometer (Perkin–Elmer, Waltham, MA, USA) at the Instituto de Macromoléculas Professora Eloisa Mano, Rio de Janeiro, Brazil. The FTIR spectra were obtained using ATR (diamond crystal) in an inert atmosphere with a resolution of 4 cm^−1^ in the range 4000–675 cm^−1^. Stored results were the averages of 124 scans.

#### 2.2.6. Low-Field Nuclear Magnetic Resonance (LF-NMR ^1^H)

The basis of NMR relaxometry is that the excited spins return from their high energy level to their original state during the relaxation of the hydrogen nucleus at different times (T_1_H). In this work, T_1_H was applied to investigate the dispersion of the nanocharge of magnetite in the polyurea matrix. For the determination of the nuclear relaxation measures, a low-field nuclear magnetic resonance spectrometer from Oxford Instruments, model Maran Ultra 0.54 T, was used, operating at a frequency of 23 MHz (for hydrogen nucleus). The spin–lattice relaxation time of the hydrogen was determined by the inversion recovery pulse sequence (180°–90°). The temperature employed was 23 °C. The amplitude (τ) was 40 points and varied between 0.1 and 5000 milliseconds (ms), with 4 measurements for each point, and a recycling interval of 1 s. The spin–spin relaxation time of the hydrogen was measured by the CPMG pulse sequence, with an interval time between the pulses of 90° and 180° of 100 microseconds. The relative relaxation intensities and values were obtained by fitting the exponential data with the help of Origin-Pro software (version 8, Originlab Corporation, Northampton, MA, USA).

#### 2.2.7. Thermogravimetric Analysis (TGA) and Scanning Differential Calorimetry (DSC)

The thermogravimetric (TGA) and differential scanning calorimetry (DSC) analyses of the samples were investigated one time using a Perkin–Elmer STA 6000. A heating rate of 20 °C/min from 30 to 300 °C and a nitrogen atmosphere with a gas flow rate of 20 mL/min were applied.

## 3. Results and Discussion

### 3.1. Crude Oil Magnetic Removal

**Figure 2:**
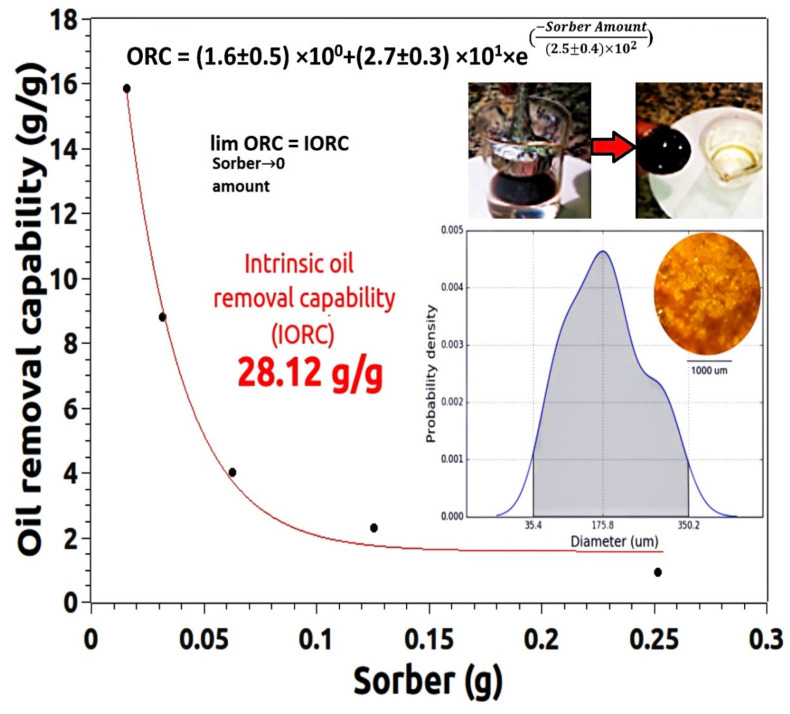
Oil removal capability (ORC) of the composited filled with three wt% of MNPs. Inset bottom right shows an optical micrograph of the tested composite as well as its grain size PDF. Inset top right shows a typical oil removal test.

Figure 2 shows the oil removal capability (ORC) of the composite sample filled with 3 wt% magnetite. This material was chosen for the test since among the composites it presents the highest magnetic force. The oil removal test followed the sequence shown in the pictures on the top right of Figure 2. In turn, the inset in the lower right corner is the PDF of the size of the particles used in this oil removal test. The optical micrograph of these particles is shown inside the PDF area.

For the oil removal test from the water surface, the petroleum was spread over the water. The nanocomposite was dispersed over the oil, and soon afterward, an Nd magnet covered with a thin aluminum foil was used to attract, trap, and remove the oil-impregnated mass.

**Figure 3:**
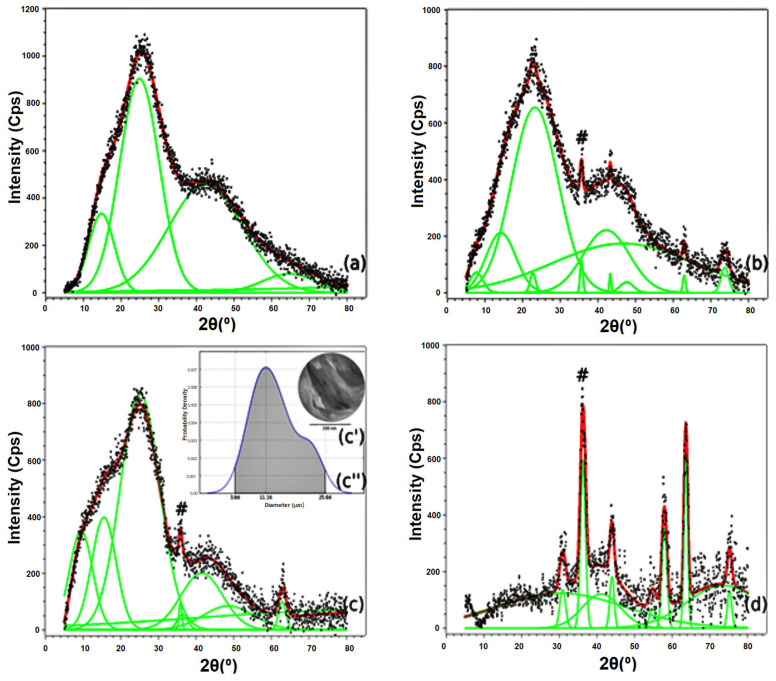
XRD Patterns of resin (**a**), composites filled with 1 wt% (**b**) and 3 wt% of magnetite (**c**) and pure magnetite (**d**). Insets (**c′**,**c″**) are the TEM and probability density function of the sample filled with 3 wt% of magnetite, respectively. The # symbol refers to the peak centered around 35°, characteristic of the magnetite particles.

**Figure 4:**
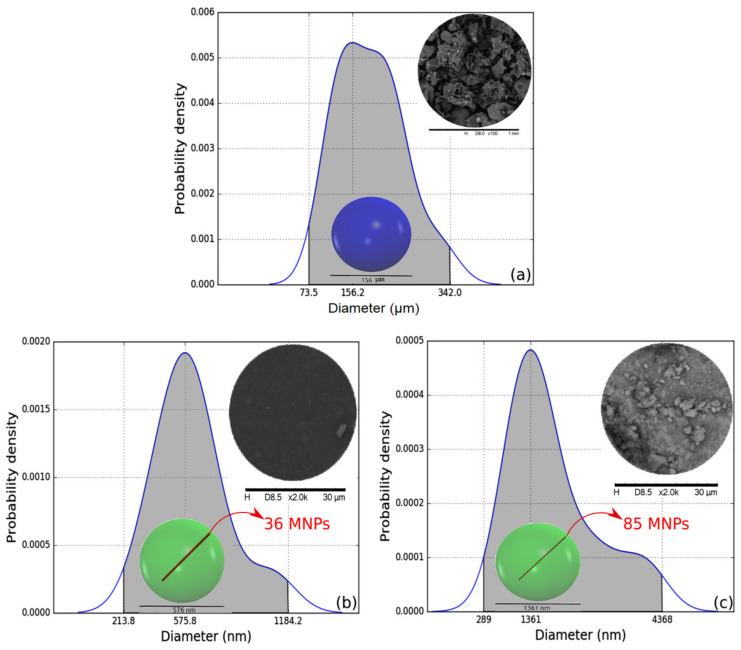
Grain size PDF functions of the resin (**a**), composite filled with three wt% of magnetite (**b**), and pure magnetite (**c**). Insets are the SEM of the samples (top right corners) and the representation of the most probable particle size (colored spheres inside the PDFs) of the polymer grains (**a**) and magnetite agglomerates (**b**,**c**). The small red spheres line inside the colored spheres (**b**,**c**) are the number of MNPs of diameter equal to 16 nm needed to fill the width of the larger objects.

More in-depth knowledge about the size and size distribution of the particles that will be used in the sorption tests is fundamental to the success of the experiment. Among the most relevant observations, the repeatability of the preparation process is key to the success of the material. Thus, the comparison of the particle size of the polymer (see Figure 3a) and the particle size of the composite used in the sorption test (see Figure 4) proves that the process of preparing the grains of the sorbing material is robust, as the results for the polymer and the composite are statistically equal.

ORC tests allow the construction of an exponential model, whose formula and parameter values are shown in Figure 2. This procedure has already been presented in detail in [39]. The limit of the ORC function tending to zero allows us to infer the value of the intrinsic oil removal capability (IORC) of the material. The IORC value of the composite is equal to 28 g of oil per gram of used composite. This is rather auspicious since it is one of the largest ever values obtained by our research group.

### 3.2. X-ray Diffraction (XRD)

Figure 3 shows the XRD patterns of the polymer, composites, and magnetite. Diffractograms were studied using a sum of Gaussian (red curves) fitting, made using Fityk software (version 1.3.1, Marcin Wojdyr, Warsaw, Poland). The Gaussians (marked in green) allow us to infer that all materials are defective, from the point of view of crystallinity. The polymer and composites have massive amorphous halos, characteristic of semi-crystalline macromolecular structures. Magnetite also presents amorphous halos, which are the result of the tiny defects commonly present in nanometric structures. Diffractograms of the composites and the magnetite also allowed for calculating, using Scherrer’s equation [44] at the peak centered around 35°, the size of the magnetite particles. The magnetite particles in the composites and the pure magnetite sample are (16 ± 1) nm in size. Among these samples, the composite filled with 3.0 wt% magnetite was studied by TEM. The obtained micrograph is shown in Figure 3c. This micrograph shows nanometric structures, corresponding to the magnetite particles inside the polymer matrix of lower electronic density. The size of these particles was determined using ImageJ software (version 1.53 m, National Institutes of Health, Bethesda, MD, USA). The results were used to build the probability density function (PDF) shown in Figure 3c. The PDF proves with a 95% probability that magnetite nanoparticles have diameters ranging from 3.6 nm to 25.6 nm. However, the most likely value is 11.2 nm. Therefore, the results of the XRD and TEM are in complete agreement and allow us to conclude that mixing the magnetite particles in the polymeric matrix does not affect the nanoparticles’ physical properties.

Figure 4 shows the PDFs of the particle sizes obtained by analyzing the SEM micrographs of the polymer, the composite filled with 3 wt% of magnetite, and the magnetite. The composite micrograph was the only one studied using SEM-BSE. This backscattered electron detector was needed due to the small number of nanoparticles used to prepare this material, making the visualization of the grains via secondary electron analysis impossible.

Figure 4a shows the PDF of the particle sizes of the polymer. The observed grains are present with a 95% probability with diameters ranging from 73.5 µm to 342 µm. Among these values, the most probable diameter size is equal to 156 µm. This last value was used to produce a translucent blue sphere with a diameter equal to 156 µm, shown inside the PDF area.

In turn, Figure 4b,c show the size of the magnetite aggregates found in the composite filled with 3 wt% of magnetite and in the sample of pure magnetite dried under vacuum. The dry magnetite presented particle sizes with a 95% probability ranging from 289 to 4368 nm. The most probable particle size value is equal to 1361 nm. This value is substantially higher than the 16 nm found for the MNPs in the XRD analysis. In practical terms, the green sphere shown inside the PDF possesses a diameter equal to 1361 nm and can hold 85 MNPs along its length. The larger size of the magnetite particles in the dry form is the result of a well-known and natural tendency to agglomerate this material on such a small scale.

Finally, the magnetite particles trapped in the polymer matrix presented sizes that with a 95% probability ranged from 214 to 1184 nm. The most probable particle size value is 576 nm. This value is about 2.4 times smaller than the most probable value found for the dried magnetite particles. This decrease in the size of the agglomerates is the result of the shearing of the magnetite during the preparation of the composite. Again, the greenish spheres represent the size of these agglomerated particles compared to the domains present in the XRD and TEM analyses. Here, the translucent greenish sphere is also shown inside the PDF. The diameter of this sphere representing the cluster of magnetite particles is equal to 576 nm, which is enough to hold up to 36 MNPs along its length. Although these particles are smaller than those in the dry magnetite sample, the higher diameter value compared to the diameters obtained by XRD and TEM indicate that the thermodynamically incompatible nature of the surfaces involved in the mixture can be further engineered so that the particle agglomeration is minimized. We leave this observation as a suggestion for future work by our research group or even other research groups concerned about improving compatibility between the phases.

### 3.3. Fourier Transform Infrared Spectroscopy Using Attenuated Total Reflectance (FTIR-ATR)

**Figure 5:**
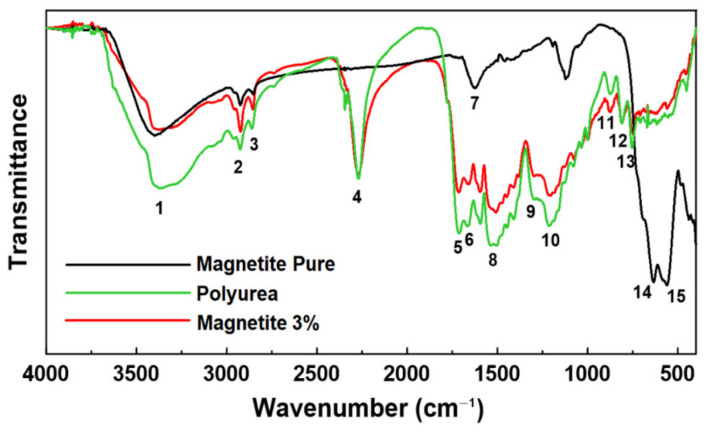
FTIR spectra of polyurea, pure magnetite, and 3% magnetite-modified composite. The main characteristic bands were identified by numbers from 1 to 15 attributed to the band assignments in Table 1.

Figure 5 shows the FTIR-ATR spectra of the polyurea, pure magnetite, and 3% magnetite-modified composite samples. The bands were labeled with numbers 1 to 15 to make their identification easier during the discussion. All the vibration modes of the FTIR spectra of the samples are presented in Table 1. It is possible to observe some important characteristic bands in Figure 5: a wide characteristic band 1 at 3400 cm^−1^, attributed to the N-H stretching; doublet characteristic bands 2 and 3 at 2924 and 2854 cm^−1^, related to the stretching of the C–H bond in the CH_2_ and CH_3_ groups; the characteristic band 4 at 2271 cm^−1^, assigned as N=C=O stretching; the characteristic band 5 at 1656 cm^−1^, assigned as C=O stretching; the characteristic band 6 at 1596 cm^−1^, assigned as NO_2_ aliphatic nitro group; the characteristic band 7 at 1622 cm^−1^, assigned as N-H bending; the characteristic band 8 at 1508 cm^−1^, assigned as NO_2_ asymmetric; the characteristic band 9 at 1260 cm^−1^, assigned as C–O–C asymmetrical stretching; the characteristic band 10 at 1203 cm^−1^, assigned as N–H bending and C–N stretching; the characteristic bands 11 and 12 at 872 and 812 cm^−1^, assigned as carbonates; the characteristic band 13 at 751 cm^−1^, assigned as alkene sp^2^ CH bending; and finally the characteristic bands 14 and 15 at 616 and 559 cm^−1^, assigned as Fe-O stretching [45,46,47,48,49,50,51,52,53]. The composite with magnetite presented less intense absorptions of the N–H group in bands 1 and 10. Similar bands were observed for the samples with and without magnetite as bands 4, 5, 6, and 9. The isocyanate band 4 was not affected by the presence of magnetite in the composite.

### 3.4. Low-Field Nuclear Magnetic Resonance (LF-NMR ^1^H)

**Figure 6:**
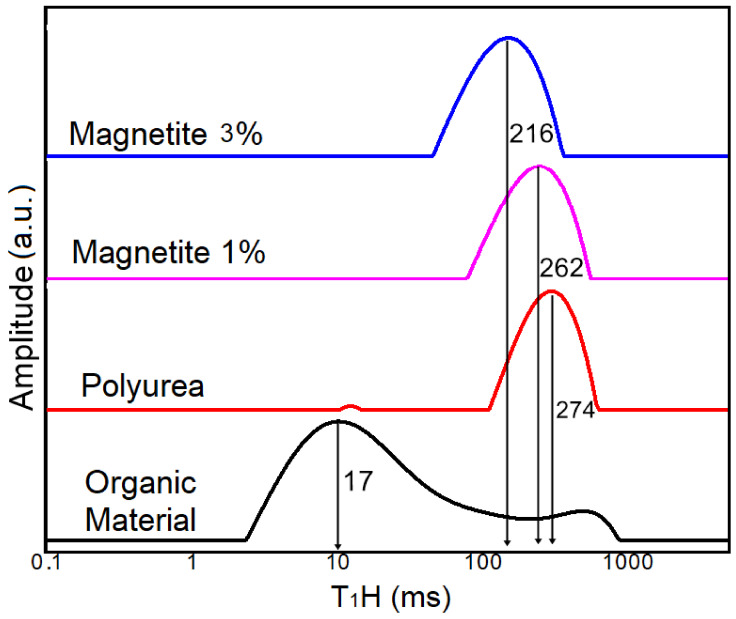
Relaxation time distribution curves (T_1_H) of polyurea and their 1% and 3% magnetite-modified composites.

The LF-NMR technique is an interesting way of characterizing polymeric materials. The values of the relaxation times T_1_ allow us to evaluate the molecular mobility of the chains in relation to each other. In this way, it is possible to distinguish between hydrogen atoms that are in different mobility domains. Thus, the LF-NMR technique made it possible to observe the effects of the nanoparticles of magnetite on the relaxation of the polyurea. Figure 6 shows the evolution of the molecular dynamics of the polymer and nanocomposites from the T_1_H values of the hydrogen atoms present in the different domains of the materials.

The polymeric matrix curve shows the T_1_H domain values at 274 ms, which is higher than the other T_1_H values of the composites. This phenomenon normally happens because the hydrogen atoms belonging to the polymer chains do not exhibit much mobility due to the tangled conformation of the long polymer chains and the high probability of interlacing between them [47,52]. On the other hand, the paramagnetism of the ferric ions (Fe^3+^) in the composite tends to influence the relaxation time of the hydrogen nucleus. Thus, the composites with 1% and 3% of magnetite registered lower T_1_H values at 262 and 216 ms, respectively. This peak displacement to the left is directly related to the effects of the nanoparticles of magnetite on the polymeric matrix and the rearrangement of the original polymer structure [54,55]. The more dispersed the magnetite is in the middle, the greater the mobility of the polymeric chains.

Thus, the H atoms present in the polymeric chain have longer relaxation times and less mobility. It is possible to observe that this curve shows one main peak related to the amorphous domain of polyurea as reported in the DRX results. In addition, a very small relaxation representation in the range of 20 ms for the same curve distribution can be observed. This representation disappeared when magnetite was added to the polyurea.

Generally speaking, when magnetite was added to the polyurea, the relaxation time was reduced, which can be attributed to the paramagnetism of the ferric ions that influences the polymeric chains by decreasing the relaxation time of hydrogen atoms leading to the greater mobility of the polymeric chains, which subsequently increases the possibility of sorption on the sorber’s available active sites.

### 3.5. Thermogravimetric Analysis (TGA)

**Figure 7:**
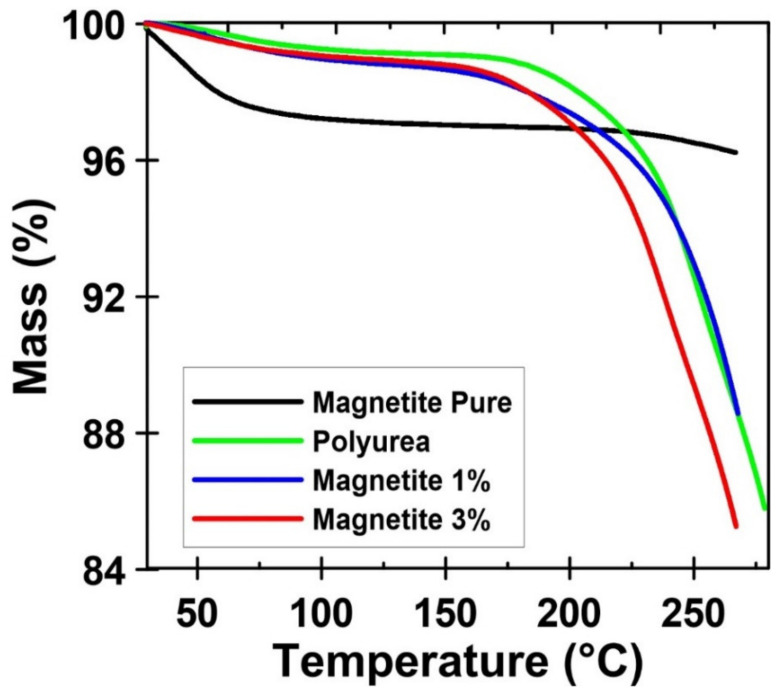
Thermogravimetric curves (TGA) of the pure magnetite, the neat polymer, and the composites with 1 and 3% of magnetite nanoparticles.

Figure 7 presents the thermogravimetric curves (TGA) of the pure magnetite, the neat polymer, and the composites with 1 and 3% of magnetite nanoparticles. The magnetite nanoparticles show typical behavior: a small initial weight loss related to the evaporation of physically adsorbed water due to the high hydrophilicity of the nanoparticles [56]. On the other hand, the macromolecular systems are completely free of water. This fact is very important due to the application of these materials as an oil sorber. The addition of nanoparticles does not seem to affect the degradation mechanism.

### 3.6. Differential Scanning Calorimetry (DSC)

Table 2 shows the enthalpy change calculated by integrating the peak area for the thermal event. DSC was used to investigate the interactions between the polymer and magnetic nanoparticles. DSC curves (not shown) reveal a first-order transition. This transition occurs naturally in the macromolecular system and becomes more intense with the increase in the number of nanoparticles.

Polymer nanocomposites consist of inorganic nanoparticles immersed in a polymeric matrix that combines the properties of both organic and inorganic precursors. When inorganic nanoparticles are incorporated into organic matrices, this allows obtaining appropriately modified polymeric materials with better thermomechanical properties and novel physical functionalities that can be adjusted by varying the composition, size, and concentration of the nanoparticulate inorganic fraction, leading to materials being specifically designed to meet the needs of different end-users [57,58,59]. So, these magnetic nanoparticles have an inorganic and polar nature; the matrix is more organic and non-polar than the nanoparticles (as was mentioned). However, due to the very small size of the particles, the surface area is huge, allowing a strong interaction between the matrix and the filler. This strong interaction naturally allows a strengthening of intermolecular forces, which makes it more difficult to increase the volume with the increase in the number of fillers. Also, the interaction between the fillers is more important at higher concentrations of nanoparticles because the inter-particle distance is decreased.

Consequently, it can be inferred that the incorporation of magnetic nanoparticles into the polymer induces changes in the polymer structure and modifies the enthalpy change, which could be associated with the interaction of the nanoparticles with the polymeric matrix [60].

## 4. Conclusions

A magnetic polymer composite has been introduced that is potentially useful for cleaning up oil spills in water. A polyurea based on an animal substrate from *Tenebrio molitor* larvae was cured with toluene diisocyanate in the presence of a magnetic powder, magnetite, which was incorporated into the final polymer material in situ. The sorber material is simple to prepare and inexpensive. The use of magnetite as a magnetic charge allowed for the efficient removal of oil from water in an amount of about 28 g of oil per gram of sorber. These results are very promising and encouraging for future environmental recovery studies involving magnetite and sustainable polymers.

The enthalpy changes increase by increasing the nanoparticles’ quantity in the composite, referring to more existing bonds compared to the neat polyurea. Based on this fact, it can be expected that there are more active sites on the nanocomposite’s surface leading to higher sorption efficiency.

When magnetite was added to the polyurea, the relaxation time was reduced which can be attributed to the paramagnetism of the ferric ions that influences the polymeric chains by decreasing the relaxation time of hydrogen atoms leading to the greater mobility of the polymeric chains, which subsequently increases the possibility of sorption on the sorber’s available active sites.

Regarding the limitations of the application of this material, its main disadvantage is the need to use biological organisms, which would be a waste of protein if cultivated for this purpose. However, as these organisms have already been used efficiently for the devulcanization of rubber, their use as a base material for preparing oil sorbents should be considered suitable and opportune.

## Figures and Tables

**Table 1:** Index of vibration modes of the FTIR spectra of polyurea, pure magnetite, and 3% magnetite-modified composite.

Characteristic Band Number	Band Assignments
1	N–H stretch amides
2	C–H stretching bond in CH_2_ and CH_3_ groups
3	
4	N=C=O (isocyanate) stretching
5	C=O & C=C conjugated stretching
6	NO_2_ aliphatic nitro group
7	N–H bending
8	Nitro compounds NO_2_ asymmetric
9	C–O–C asymmetrical stretching
10	N–H bending and C–N stretching
11	Carbonates
12	
13	Alkene sp^2^ CH bending
14	Fe-O stretching splitted bands
15	

**Table 2:** Enthalpy change calculated by integrating the peak area due to interactions between the polymer and magnetic nanoparticles.

Sample	ΔH (J/g)	Error
Polyurea	−33.6	4.29
Magnetite 1%	−51.6	6.59
Magnetite 3%	−70.6	9.02
Magnetite Pure	−65.7	8.39
